# Tigecycline Absorption Improved by Selected Excipients

**DOI:** 10.3390/ph16081111

**Published:** 2023-08-05

**Authors:** Hubert Ziółkowski, Kalina Szteyn, Dawid Jędrzkiewicz, Bartosz Rasiński, Jerzy Jaroszewski

**Affiliations:** 1 Department of Pharmacology and Toxicology, Faculty of Veterinary Medicine, University of Warmia and Mazury in Olsztyn, Oczapowskiego 13, 10-718 Olsztyn, Poland; szteyn@gmail.com (K.S.); jerzyj@uwm.edu.pl (J.J.); 2 Department of Chemistry, University of Wrocław, F. Joliot-Curie 14, 50-383 Wrocław, Poland; dawid.jedrzkiewicz@chem.uni.wroc.pl; 3 Waters Spółka z Ograniczoną Odpowiedzialnością, Wybrzeże Gdyńskie 6B, 01-531 Warszawa, Poland; bartosz_rasinski@waters.com

**Keywords:** tetracyclines, tigecycline, intestinal absorption, pharmacokinetics, excipients

## Abstract

To investigate the effects of (2,6-di-O-methyl)-β-cyclodextrin (DM-β-CD), (2-hydroxypropyl)-β-cyclodextrin (HP-β-CD), tocopherol polyethylene glycol 1000 succinate (TPGS), sodium desoxycholate (SDOCH), trimethyl chitosan (TMC), and sodium caprate (C10) on the plasma concentration and the oral bioavailability of tigecycline in broiler chickens. To test the effects of the excipients on absorption of tigecycline, a tetracycline that is poorly absorbed from the gastrointestinal tract, broiler chickens were used as an animal model. Tigecycline (10 mg/kg body weight) was administered intravenously, orally, and orally with one of the excipients. Plasma samples were taken after administration. To measure tigecycline concentrations, high-performance liquid chromatography coupled with tandem mass spectrometry was used. Compartmental and non-compartmental analyses were used for pharmacokinetic analyses of mean plasma concentrations versus time. With the exception of sodium caprate, all the excipients significantly increased the area under the curve and bioavailability of tigecycline (*p* < 0.05). These parameters were approximately doubled by HP-β-CD, TPGS, and SDOCH, with 95% confidence intervals (95% CIs) for the difference that included only increases of 1.5-fold or higher (bioavailability: control, 1.67%; HP-β-CD, 3.24%; TPGS, 3.30%; and SDOCH, 3.24%). The increases in these parameters were smaller with DM-β-CD and TMC (DM-β-CD, 2.41%; TMC, 2.55%), and the 95% CIs ranged from close to no difference to nearly double the values in the control group. These results indicate that HP-β-CD, TPGS, and SDOCH substantially increase the area under the curve and oral bioavailability of tigecycline. They suggest that DM-β-CD and TMC may also substantially increase these parameters, but more research is needed for more precise estimates of their effects.

## 1. Introduction

Currently, tetracyclines are one of the most commonly used antimicrobials in veterinary medicine. Additionally, in human medicine, they serve as invaluable drugs for infections with atypical microorganisms. These facts result from their broad spectrum of action, their high efficacy, and the ease with which they are inexpensively synthesized at industrial scale [1].

The pharmacokinetics of these drugs vary widely between tetracyclines and species; for example, the bioavailability of minocycline is over 90%, whereas that of meclocycline [2] and tigecycline is less than 1%. It is generally thought that trans-membrane tetracycline transport (and thus, absorption and distribution of these drugs) is regulated by simple diffusion, which is probably affected by the lipophilicity of these substances [3,4]. However, it seems that the absorption and distribution of tetracyclines does not always correlate well with diffusion, which suggests that these pharmacokinetic parameters can be strongly affected by factors other than the lipophilicity of these drugs. This is particularly relevant because most tetracyclines in human and animal medicine are administered orally, with the exceptions of tigecycline and eravacycline [2,5]. Although this method of administration has obvious benefits, its major drawback is the widely differing pharmacokinetics of these drugs, particularly their bioavailability [6].

Our recent study has shown that one of the causes of these differences in oral bioavailability may be active efflux of tetracyclines from enterocytes [7]. Out of all known “efflux pump” proteins, p-glycoprotein (P-gp) and breast cancer resistance protein (BCRP) make the largest contribution to the active removal of xenobiotics from cells [8]. The concentration of these transmembrane proteins is very high in epithelium, which is found in all kinds of biological barriers, including intestinal barriers [9].

In a previous study, we showed that blocking efflux pumps with cyclosporine A substantially increases the oral bioavailability of six tetracyclines, thus demonstrating that it is possible to efficiently modify the absorption of these drugs [7]. Currently, efflux pumps like pg-P and BCRP are blocked by administering a number of pharmacologically active substances, e.g., verapamil, ketoconazole, ritonavir, cyclosporin A, etc. [10]. Nevertheless, the pharmacological activity of these substances can cause effects that should be avoided, such as immunosuppression or changes in the circulatory system. In an attempt to avoid these harmful effects, work is underway on the design of specific blockers of efflux pumps such as valspodar, zosuquidar, encequidar, and ONT-093. However, these substances are still being studied and only a few have reached the stage of preliminary clinical trials, e.g., encequidar [11].

Interestingly, the excipients that are used to formulate most oral medications may be promising alternatives to the above-mentioned efflux-pump blockers. Excipients that are used to dissolve oral and/or injectable drugs include pH modifiers, water-soluble organic solvents, oils, surfactants, water-insoluble organic solvents, medium-chain and long-chain triglycerides, cyclodextrins, and phospholipids [12]. To date, the primary purpose of these excipients has been to allow proper administration, to facilitate manufacture, to increase the stability of formulations, to improve aesthetics, or to facilitate identification of medications [13]. However, it should be kept in mind that excipients can interact with drugs, affecting their absorption and bioavailability [14,15]. The mechanisms of these interactions are not fully determined. Some excipients affect cellular connections, e.g., tight junctions [16], others increase drug solubility [12] or serve as surfactants [17], and others interact with protein transporters like efflux pumps [18,19]. Thus, excipients can improve the oral bioavailability of drugs [15,20].

The results of our previous study strongly suggest that efflux pumps in the intestinal epithelium help to regulate tetracycline absorption from the gastrointestinal tract [7]. This led us to hypothesize that the ability of excipients to modify absorption, and particularly their interactions with efflux pumps, may be useful for improving the oral bioavailability of tetracyclines. A particularly challenging case with which to test this hypothesis would be tigecycline, which is so poorly absorbed from the gastrointestinal tract that it is usually administered intravenously [21]. This antimicrobial is the first approved antibiotic from the class of glycylcyclines [5], and it is derived from minocycline by adding a tert-butyl-glycylamido side chain to carbon 9 of the D ring of the TC nucleus [22]. In human and veterinary medicine, tigecycline has a range of useful applications, including complicated skin and skin structure infections and complicated intra-abdominal infections caused by Gram-positive and negative aerobic bacteria [23,24]. More importantly, however, an improvement in tigecycline absorption via co-administration of excipients would suggest that the oral bioavailability of other tetracyclines could be similarly improved, thus reducing doses and the resulting amount of the drugs that is excreted into the environment [25].

Therefore, the objective of this study was to evaluate the effect of excipients on the oral bioavailability of tigecycline, which appears to be affected by efflux-pump modulators. To achieve this objective, six excipients that may block efflux pumps were selected, (2,6-di-O-methyl)-β-cyclodextrin (DM-β-CD) [26], (2-hydroxypropyl)-β-cyclodextrin (HP-β-CD) [27], tocopherol polyethylene glycol 1000 succinate (TPGS) [28], sodium desoxycholate (SDOCH) [29], trimethyl chitosan (TMC) [30], and sodium caprate (C10) [29]. The effects of these excipients on the bioavailability of tigecycline in broiler chickens, which we successfully used as a model in a previous study, were then investigated.

## 2. Results

**Figure 1:**
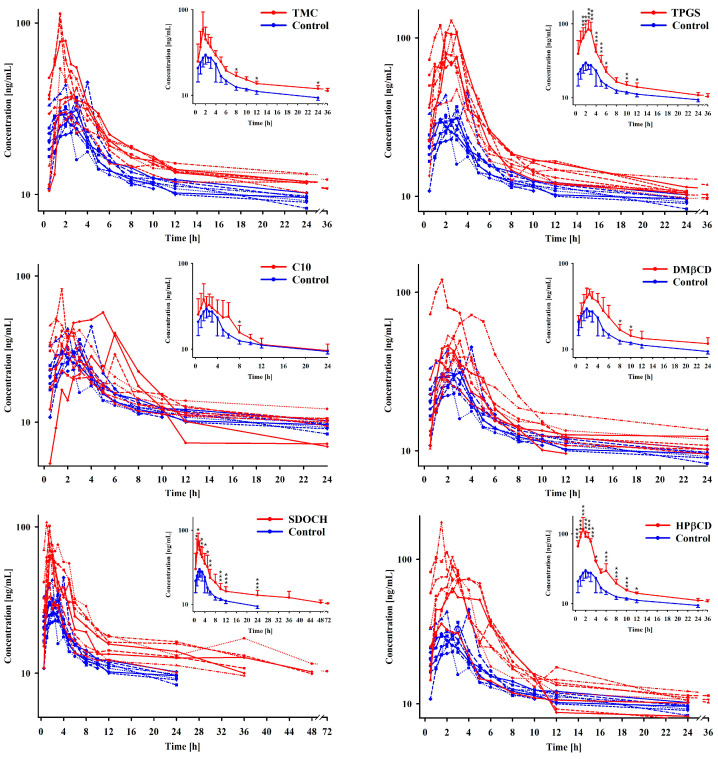
Individual and mean (±SD; inserts) plasma concentrations of tigecycline (10 mg/kg BW) after oral administration with excipients: TMC—trimethyl chitosan; C10—sodium caprate; SDOCH—sodium desoxycholate; TPGS– tocopherol polyethylene glycol 1000 succinate; DM-β-CD—(2,6-di-O-methyl)-β-cyclodextrin; and HP-β-CD—2-hydroxypropyl)-β-cyclodextrin. * Significantly different at *p* < 0.05; ** significantly different at *p* < 0.01; *** significantly different at *p* < 0.001.

All of the excipients increased the AUC_0→t_ and the bioavailability of tigecycline, and these increases were statistically significant, with the exception of the effects of C10 (Figure 1). Note that, although the differences between C10 and the control was not statistically significant, the 95% CI for the difference in bioavailability extends from –0.34 to 1.09% (absolute difference). Also note that the 95% CIs for the bioavailability differences between DM-β-CD and TMC and the control extend from 0.02 to 1.46% and 0.16 to 1.60%, respectively.

**Figure 2:**
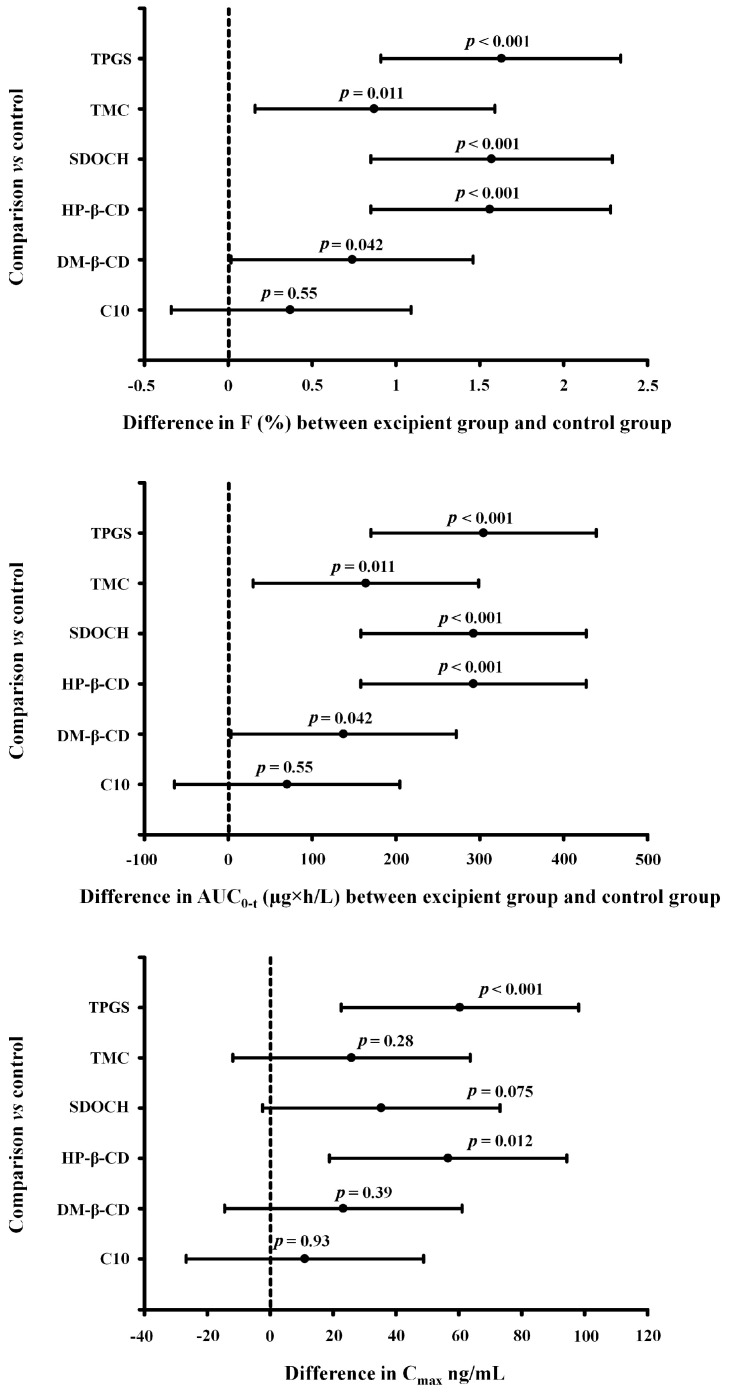
The 95% confidence intervals for the difference for bioavailability (F), area under the concentration–time curve calculated from 0 to t (AUC_0→t_) and maximum plasma concentrations (C_max_). C10−sodium caprate; DM−β−CD−(2,6−di−O−methyl)−β−cyclodextrin; HP−β−CD−2−hydroxypropyl)−β−cyclodextrin; SDOCH−odium desoxycholate; TMC−trimethyl chitosan; and TPGS−tocopherol polyethylene glycol 1000 succinate.

On the other hand, the 95% CIs for the bioavailability differences between the other three excipients and the control all have lower limits of 0.85% or higher. The 95% CIs for the differences in AUC_0-t_ display patterns that are similar to those for the differences in bioavailability (Figure 2).

Although only the effects of HP-β-CD and TPGS were significant, all of the excipients increased the C_max_ of tigecycline (Figure 1, Table 1). With TPGS and HP-β-CD administration, C_max_ was about 2.5-times higher than in the control group. Additionally, C_max_ was almost two times higher when SDOCH was administered, and the 95% CI for the difference extended from –2.43 to 73.09 (*p* = 0.07) (Figure 2).

Although the SDOCH, TPGS, and HP-β-CD significantly increased the plasma drug concentrations at nearly all sampling points (Figure 1), the bioavailability, and the AUC_0-t_, they did not significantly influence the absorption time parameters (k_ab_, t_1/2kab_, MAT) or the elimination time parameters (k_1/2β_, t_1/2β_). Also, there was no significant influence on MRT (Table 1).

## 3. Discussion

The results of our study indicate that five of the six tested excipients increase the AUC_0→t_ and oral bioavailability of tigecycline, thus raising its plasma concentrations, and they suggest that C10 may also increase these values. The 95% CIs for the differences in bioavailability indicated that HP-β-CD, SDOCH, and TPGS increase the bioavailability by at least as much as 0.85% (absolute difference), which is a substantial increase in bioavailability. However, it is unclear whether DM-β-CD and TMC increase bioavailability to a clinically relevant extent, as the lower 95% confidence limits for the absolute difference are at 0.02 and 0.16% difference, respectively. Finally, the 95% CI for C10 suggests that it might even decrease the bioavailability of tigecycline. Thus, more research would be needed to obtain more precise estimates of the effects of DM-β-CD, TMC, and C10. In particular, sample sizes of 32 birds per group or larger would greatly improve the precision because the width of the 95% CIs would be half of the ones in the present study. Alternatively, a less-expensive option would be to combine the results of several smaller studies via meta-analytic techniques.

The small differences in absorption and elimination time, which were not statistically significant, indicate that the AUC_0→t_ and bioavailability values can be directly compared. The increases in the absorption parameters led to increases in the AUC/MIC, which is a pharmacodynamic index used to predict clinical outcomes and establish clinical breakpoints for tetracyclines [5].

Although the excipients used in this study may modify absorption by various mechanisms, they all block efflux pumps [18,29]. Additionally, our previous study indicates that the pharmacokinetics of tetracyclines are affected to a greater extent by interactions between these drugs and efflux pumps than by other interactions [7]. The level of efflux pump expression in epithelium, which lines the gastrointestinal tract, is high [9], supporting the idea that these pumps play an important role in the regulation of the absorption of these drugs from the gastrointestinal tract. Nevertheless, we cannot rule out the possibility that these excipients also dilate tight junctions or affect the lipid elements of cell membranes. However, it seems possible that the main mechanism of their effect on tigecycline bioavailability is via interactions with efflux pumps in the epithelium of the gastrointestinal tract because this is the only mechanism that all of the excipients have in common. Additionally, we reduced the possible influence of other mechanisms, such as effects on solubility, by using a tigecycline form with a solubility in water that is several orders of magnitude higher than that of the base forms of tigecycline.

The present results are consistent with the results of other authors who used similar excipients in their studies with anthracyclines, which are derivatives of the tetracycline family and should have similar absorption mechanisms to those of other tetracyclines. For example, Lo and Huang [29] reported that treatment with SDOCH and C10 significantly increases epirubicine accumulation in Caco-2 cells, apical to basolateral absorption of epirubicin across Caco-2 monolayers, and mucosal to serosal absorption of epirubicin in rat jejunum and ileum. Furthermore, Tilloy et al. [31] found that co-administration of methylated cyclodextrins substantially increased doxorubicin transport through the blood–brain barrier in vitro. Similarly, Dintaman and Silverman [28] reported that in vitro TPGS administration improved the accumulation of doxorubicin in cells, and Zare et al. [30] reported that chitosan increased the transport of this anthracycline across the intestinal wall. The authors of all these studies suggested that the effects they observed were partially or mainly due to efflux modulation, e.g., blocking p-glycoprotein.

Pharmacokinetically speaking, a drawback of tetracyclines is that, in the gastrointestinal tract, they are subject to various types of interactions, especially with divalent cations [32] and food [33], which decrease their oral bioavailability and plasma concentrations. Although the interactions of tetracyclines with efflux pumps complicate their pharmacokinetics, especially their absorption, these interactions suggest a possible method of modifying the kinetics of these drugs to improve their oral bioavailability.

Indeed, the results of the present study clearly indicate that improvement of the oral bioavailability of tigecycline is possible with the use of common pharmaceutical industry substances that have no observed pharmacological properties or ones with little clinical importance. Of the excipients that were used in previous studies [34] and this study, HP-β-CD and TPGS are additives to orally used drugs [35,36], SDOCH is an inactive ingredient for intravenous use [37,38], C10 is a food additive [39], and DM-β-CD can be used as a dermal, rectal, or oral ingredient according EMA regulations [40]. Each of these excipients has a different influence on absorption, and even if the present authors’ thesis about efflux modulation is incorrect, the fact remains that these excipients substantially increased the oral bioavailability of tigecycline, the least absorbable of the tetracyclines, which shows their potential for modulating the oral absorption of tetracyclines. Finally, because these excipients make it possible to increase blood concentrations of tetracyclines after oral administration while maintaining the same dose, they could help to limit increases in tetracycline consumption, so that a smaller amount of the drugs is released into the environment, thus reducing their contribution to the development of drug resistance.

One limitation of our study is that it did not include advanced formulations of tigecycline with excipients like nanoparticles, micelles, complex formulations, etc. Nevertheless, this study shows that the absorption of tigecycline, and therefore absorption of other tetracyclines too, are very susceptible to modification. Moreover, it is possible that, when special formulations of tetracyclines using some of the studied excipients are prepared, the increase in bioavailability could be even higher than what was observed. Thus, it is particularly interesting that attempts are currently underway to combine these excipients with each other and then with anthracyclines to further improve the absorption and distribution of these drugs. A good example is TPGS, which has been conjugated with cyclodextrin [41] or chitosan [42] and then with doxorubicin. Thanks to this composition, the efflux was significantly decreased in cancer cell lines [41,42] and animal models [41]. Transferring this approach to tetracyclines may significantly improve the use of these drugs in the near future, and the latest findings lend support this prediction [43].

## 4. Materials and Methods

### 4.1. Chemicals and Reagents

Tigecycline, in the form of a powder for preparing solutions for infusion (Tygacil), was obtained from Pfizer (New York, NY, USA), whereas tigecycline-d9, which served as an internal standard (IS) for tigecycline, was obtained from Toronto Research Chemicals (North York, ON, Canada). Five excipients, DM-β-CD (MW = 1331.35 g/mol), HP-β-CD (MW = 1541.54 g/mol), TPGS (MW = 662.9 g/mol), SDOCH (MW = 414.6 g/mol), and C10 (MW = 194.25 g/mol), were purchased from Sigma-Aldrich (St. Louis, MO, USA), whereas TMC (239.70 g/mol) was prepared according to Sieval et al. (1998) [44]. Chemicals for chromatography, i.e., 1,2-dichloroethane, acetonitrile, formic acid, and water, were obtained from Sigma-Aldrich (St. Louis, MO, USA).

### 4.2. Animals

Sixty-four healthy Ross broiler chickens (age: 3 weeks) were acquired from a commercial farm (WIMAR, Stawiguda, Poland) and transported to the vivarium in the Faculty of Veterinary Medicine at the University of Warmia and Mazury in Olsztyn, Poland. Male and female chickens were used as there is no evidence that sex influences the studied phenomena. The vivarium was air-conditioned, which allowed the temperature and relative humidity to be maintained at 22 °C and 45–65%, respectively. The same light–dark cycle was used as at the commercial farm (16 h and 8 h, respectively). The birds were observed throughout a 1-week acclimatization period, during which they were all fed a standard broiler growth diet (drug-free) with ad libitum access to water and did not receive any pharmacological treatment. On the day the experiment began, the animals were 4 weeks old, with a mean body weight (BW) of 1.75 ± 0.19 kg. During the experiment, no clinical signs of disease were observed. The Local Ethics Committee in Olsztyn registered and approved this study (Ethics Committee Opinion No. 44/2016).

### 4.3. Experimental Design

Here, we use the same “chicken model” for pharmacokinetics studies focused on the effects of efflux pumps that has been successfully used by our team and others in previous studies [7,45]. We use this model because efflux pumps/proteins are abundant in chicken guts [46], the expression pattern of these pumps is similar in human and chicken guts [47], and efflux pumps are very well conserved across species. An additional advantage of this experimental design is that it is possible to define the pharmacokinetic profile of tigecycline for each animal when using chickens, unlike rodent models, thus reducing the number of animals in the study, which is consistent with the ethical precept of using as few animals as possible.

The broilers were randomly divided into eight groups: one for intravenous administration (to calculate absolute bioavailability, total body clearance, and volume of distribution) and seven groups for oral administration, with eight birds in each group. All animals in each group were administered tigecycline at 10 mg/kg BW. To test the hypothesis that some excipients will change tigecycline absorption, the excipients were orally administered to six groups (dissolved in water) together with tigecycline, and the results were compared to oral administration without excipients. The excipient doses were as follows: TMC—50 mg/kg [48], DM-β-CD—113.6 mg/kg [26], HP-β-CD—300 mg/kg [49], TPGS—50 mg/kg [50], SDOCH—500 mg/kg [51], and C10—50 mg/kg [52]. The differences in excipient doses were due to their different chemical structures and safety profiles.

Due to the differences in the body weight of the animals, the solutions with excipients were prepared individually in a volume of 4 mL. Each excipient was weighed out according to the indicated dosage (see previous paragraph) for the body weight of an individual animal and then dissolved in 4 mL of water. Then, an appropriate volume of tigecycline was taken from a stock solution with a concentration of 50 mg/mL and added to the excipient solution. Thus, the final volume for each animal was ~4.4 mL, with slight variations in order to maintain the appropriate dose of tigecycline for the weight of each bird.

Feed was withheld from 6 h before until 3 h after tigecycline administration, and water was withheld from 1 h before until 1 h after. For minimization of any potential interactions during absorption, the birds received only analytical-grade compounds (except tigecycline), which were dissolved in deionized water. The drugs were orally administered to the animals via gastric tube gavage or intravenously administered via a 26 G venflon cannula (0.6 × 20 mm) in the left brachial vein.

In the intravenous administration groups, samples were collected from the right brachial vein at 0 h, then at 0.083 h, and 0.25 h using heparinized tubes, and the same procedure was used for all groups at 0.5, 1, 1.5, 2, 2.5, 3, 4, 5, 6, 8, 10, 12, 24, 36, 48, and 72 h after drug administration. For plasma separation, the samples were centrifuged at 1650× *g* and 4 °C for 10 min, and then the plasma was stored at −70 °C until analysis.

### 4.4. Chromatography and Sample Preparation

For tigecycline determination, plasma samples were prepared and high-performance liquid chromatography with tandem mass spectrometry (HPLC-MS/MS) was performed according to the method of [53], which was slightly modified and re-validated for chickens [6]. To 250 µL of plasma thawed at room temperature, 10 µL of IS working solution was added. Immediately after the addition of IS, the samples were shaken at 1000 rpm for 5 s. After protein precipitation with 1 mL of acetonitrile, the samples were shaken at 3000 rpm for 15 s and centrifuged at 2250× *g* for 10 min at 4 °C. Next, the samples were extracted with 1.5 mL of 1,2-dichloroethane, shaken at 3000 rpm for 30 s, and centrifuged at 2250× *g* for 10 min at 4 °C. Finally, 150 µL of the water fraction was filtered (0.22 µm) and injected into the HPLC-MS/MS system.

### 4.5. Pharmacokinetic Analysis

Based on the chromatographic analysis of plasma concentrations of tigecycline in each individual, data on plasma concentrations versus time were analyzed using ThothPro™ software (Gdańsk, Poland). Non-compartmental analysis of both routes of administration was performed and included the area under the concentration-time curve calculated from 0 to t (AUC_0→t_) and from 0 to infinity (AUC_0→∞_) according to the linear trapezoidal rule, the elimination rate constant (β), and the half-life in the elimination phase (t_1/2β_). Mean residence time from 0 to t (MRT_0→t_) and from 0 to infinity (MRT_0→∞_) was calculated based on AUC_0→t_ and AUC_0→∞_ as well as the area under the first moment of the curve from 0 to t (AUMC_0→t_) and 0 to infinity (AUMC_0→∞_). In the intravenous group, the following were also determined: apparent volume of distribution (Vd_area_), based on AUC_0→t_ (non-compartmental analysis); steady-state volume of distribution (Vd_ss_) (non-compartmental analysis); and total body clearance (Cl_B_). Furthermore, in all oral groups, the absorption rate constant (k_ab_) was calculated. The mean absorption time (MAT) and half-life in the absorption phase (t_1/2kab_) were calculated using the single-compartment first-order process [54]:

$$MAT=1/k_{ab}$$

$$t_{1/2kab}=0.693/k_{ab}$$

The maximum and the last plasma concentrations (C_max_ and C_last_, respectively) and the time (t_max_) of C_max_ and C_last_ after oral administration of the drugs were determined individually for each animal and were expressed as mean values (±SD). In turn, after intravenous administration, C_max_ and t_max_ were the first determined concentration and the time of the first determined concentration (C_0.083_ and t_0.083_, respectively), respectively, and were also expressed as mean values (±SD). To calculate the value of the absolute bioavailability (F), the following equation was used:

$$F={{(AUC}_{0\to t}}_{oral\;individual}/{{AUC}_{0\to t}}_{intravenous\;mean})\times 100\%$$

### 4.6. Statistical Analysis

The results of pharmacokinetic and HPLC-MS/MS analysis were analyzed by one-way ANOVA followed by Dunnett’s test for calculation of *p*-values, with *p* < 0.05 regarded as statistically significant. Additionally, for selected pharmacokinetic parameters, the 95% confidence intervals (95% CIs) from Dunnett’s test are also provided. These calculations were performed with R, version 4.2.2.

## 5. Conclusions

In conclusion, the results of this study indicate that co-administration of DM-β-CD, HP-β-CD, TPGS, SDOCH, and TMC increases the absorption of tigecycline. It is possible that these increases could be related to the efflux modulation because all of these excipients interact with efflux pumps. In a previous study, we have shown that administration of a non-specific efflux pump blocker significantly improves the absorption of tetracyclines. However, these ideas about efflux modulation are only speculation at this point, and further studies should evaluate whether efflux pumps are involved in tetracycline dispositions and to what extent they are involved. Although tigecycline is not typically administered orally due to its poor absorption, these results have valuable implications for the use of other tetracyclines. From a pharmaceutical point of view, when these excipients are used to prepare tetracycline formulations, the possibility that they will increase the absorption of the drugs should be considered. From a pharmacological point of view, when using well-absorbed tetracyclines such as minocycline, doxycycline, or tetracycline, it should be considered that these excipients may significantly increase their concentrations in the blood, thus increasing their antimicrobial effectiveness at a lower dose.

## Figures and Tables

**Table 1:** Pharmacokinetic parameters (mean ± SD) of tigecycline (10 mg/kg BW) after oral (PO) and oral with excipient administration in broiler chickens (*n* = 8).

Pharmacokinetic Parameters	Tigecycline (Control)	Tigecycline + Trimethyl Chitosan	Tigecycline + Sodium Caprate	Tigecycline + Sodium Desoxycholate	Tigecycline + Tocopherol Polyethylene Glycol 1000 Succinate	Tigecycline + (2,6-di-O-methyl)-β-Cyclodextrin	Tigecycline + (2-Hydroxy propyl)-β-Cyclodextrin	Tigecycline Intravenous	
AUC_(0-t) _ (µg×h/L)	312.28 ±52.29	476.40 * ±104.53	382.49 ±42.32	604.84 *** ±131.16	616.79 *** ±86.13	449.82 * ±81.52	604.70 *** ±157.46	18,682.07 ±3219.19	
AUC_(0-∞)_ (µg×h/L)	849.92 ±280.50	1158.50 ±372.069	1065.04 ±533.03	1492.16 ±407.07	1316.84 ±318.41	1254.72 ±407.82	1261.21 ±408.61	20,069.14 ±3089.97	
β (h^−1^)	0.029 ±0.033	0.022 ±0.015	0.022 ±0.014	0.014 ±0.003	0.018 ±0.009	0.017 ±0.006	0.018 ±0.01	0.015 ±0.006	
t_1/2β_ (h)	39.44 ±16.56	42.81 ±16.17	46.3 ±32.12	53.13 ±10.13	46.92 ±20.77	48.31 ±24.06	45.18 ±23.39	53.88 ±25.31	
C_max_ (µg/mL)	0.035 ±0.006	0.061 ±0.033	0.046 ±0.018	0.071 ±0.029	0.096 * ±0.028	0.058 ±0.027	0.091 * ±0.043	28.61 ±9.84	
t_max_(h)	2.50 ±0.80	2.06 ±0.56	2.63 ±1.89	1.50 ±0.27	2.50 ±0.53	2.57 ±0.77	2.44 ±0.82	0.083	
C_last_ (µg/mL)	0.010 ±0.001	0.011 ±0.002	0.010 ±0.002	0.011 ±0.003	0.011 ±0.001	0.010 ±0.003	0.010 ±0.001	0.019 ±0.003	
t_last_(h)	22.25 ±0.49	27.0 ±8.49	24.0	39.0 ±15.38	30.0 * ±6.42	24	28.50 ±6.21	96	
AUMC_(0-t)_ (mg×h×h/L)	2897.21 ±832.68	5239.9 ±2466.15	3483.17 ±312.98	8516.48 ±3295.12	6175.31 ±2115.48	3907.29 ±483.96	5663.75 ±2426.45	220,673.25 ±48,869.82	
AUMC_(0-∞)_ (mg×h×h/L)	51,143.95 ±28,612.95	73,317.77 ±44,132.72	86,998.69 ±107,239.9	115,013.7 *** ±51,553.9	83,919.44 * ±59,131.27	88,961.75 ±93,875.95	78,914.06 * ±62,095.32	477,072.60 ±119,564.68	
MRT_(0-t)_ (h)	9.04 ±1.85	10.57 ±3.54	9.16 ±0.87	14.63 * ±4.33	9.89 ±2.72	8.81 ±1.07	9.11 ±1.84	11.93 ±2.77	
MRT_(0-∞)_ (h)	53.82 ±22.32	56.93 ±24.09	62.03 ±25.88	70.37 ±13.51	57.85 ±29	62.83 ±35.84	54.71 ±33.07	24.36 ±7.85	
k_ab_ (h)	0.97 ±0.54	1.21 ±1.25	0.85 ±0.91	1.29 ±0.68	0.74 ±0.54	1.17 ±0.67	0.78 ±0.76	Cl (L/h*kg)	0.41 ±0.08
t_1/2kab_ (h)	0.91 ±0.77	1.73 ±1.90	1.50 ±0.97	0.76 ±0.52	1.81 ±2.07	1.02 ±1.03	1.54 ±0.97	Vd_area_ (L/kg)	32.22 ±17.20
MAT (h)	1.32 ±1.12	2.50 ±2.74	2.46 ±1.40	1.10 ±0.76	2.61 ±2.99	1.43 ±1.51	2.23 ±1.40	Vd_ss_ (L/kg)	4.98 ±1.98
F (%)	1.67 ±0.27	2.55 ** ±0.56	2.04 ±0.23	3.24 *** ±0.70	3.30 *** ±0.46	2.41 * ±0.43	3.24 *** ±0.84	-	-

AUC_0→t_—area under the concentration vs. time curve from 0 to t; AUC_0→∞_—area under the concentration vs. time curve from 0 to ∞; β—slope of the elimination phase; t_1/2β_—half-life in the elimination phase; C_max_—maximum plasma concentration (for intravenous it is first measured plasma concentration); t_max_—time of maximum concentration (for intravenous it is first time of measured concentration); C_last_—last measured plasma concentration; t_last_—time of last measured concentration; AUMC_0→t_—area under the first moment curve; AUMC_0→∞_—area under the first moment curve from 0 to ∞; MRT_0→t_—mean residence time; MRT_0→∞_—mean residence time from 0 to ∞; k_ab_—absorption rate constant; t_1/2kab_—half-life in the absorption phase; MAT—mean absorption time; F—absolute bioavailability; Cl_B_—total body clearance; Vd_ss_—volume of distribution in steady state; and Vd_area_—apparent volume of distribution. * Significantly different from control at *p* < 0.05. ** Significantly different from control at *p* < 0.01. *** Significantly different from control at *p* < 0.001.

## Data Availability

Data is contained within the article.
